# Odor Discrimination as a Marker of Early Alzheimer’s Disease

**DOI:** 10.3233/JAD-230077

**Published:** 2023-08-01

**Authors:** Egle Audronyte, Gyte Pakulaite-Kazliene, Vaiva Sutnikiene, Gintaras Kaubrys

**Affiliations:** Clinic of Neurology and Neurosurgery, Institute of Clinical Medicine, Faculty of Medicine, Vilnius University, Vilnius, Lithuania

**Keywords:** Alzheimer’s disease, mild cognitive impairment, olfaction, olfactory impairment

## Abstract

**Background:**

Olfactory dysfunction is an early symptom of Alzheimer’s disease (AD). However, olfactory tests are rarely performed in clinical practice because their diagnostic efficacy in detecting early AD is unclear.

**Objective:**

To investigate odor discrimination in patients with early AD and the efficacy of olfactory discrimination tests in differentiating these patients from subjects with normal cognition (CN).

**Methods:**

Thirty patients each with mild dementia due to AD (MD-AD) and mild cognitive impairment due to AD (MCI-AD) and 30 older subjects with CN were enrolled. All participants underwent cognitive examinations (CDR, MMSE, ADAS-Cog 13, and verbal fluency) and odor discrimination tests (Sniffin’ Sticks test, Burghart®, Germany).

**Results:**

The MD-AD group achieved significantly worse scores on the olfactory discrimination test than the MCI-AD group, and the MCI-AD group achieved significantly worse results than the CN group (*p* < 0.05). A cut-off score of≤10 had a diagnostic accuracy of 94.44% (95% CI, 87.51–98.17%) in differentiating patients with MCI-AD/MD-AD from subjects with CN and of 91.67% (95% CI, 81.61–97.24%) in differentiating those with MCI-AD from subjects with CN. Our multinomial logistic regression model with demographic data and ADAS-Cog 13 scores as predictor variables correctly classified 82.2% of the cases (CN, 93.3%; MC-AD, 70%; MD-AD, 83.3%); on adding the olfactory discrimination score to the model, the percentage increased to 92.2% (CN, 96.7%; MCI-AD, 86.7%; MD-AD, 93.3%).

**Conclusion:**

Odor discrimination is impaired in cases of early AD and continues to deteriorate as the disease progresses. The olfactory discrimination test showed good diagnostic efficacy in detecting early AD.

## INTRODUCTION

In 2019, more than 55 million persons were estimated to have dementia worldwide [1]. However, most patients remain undiagnosed. According to the estimates of Alzheimer’s Disease International, up to 75% of the patients worldwide and up to 90% in low- and middle-income countries are not diagnosed [2]. The prevalence of dementia is continuously increasing and is estimated to reach 139 million cases by 2050 [1, 2]. Considering the present difficulties with accurate diagnostics and the increasing prevalence of dementia, identifying affordable and widely accessible diagnostic markers is of major importance.

Alzheimer’s disease (AD) is the most common cause of dementia, accounting for up to 70% of all dementia cases [3]. Currently used biomarkers for diagnosing AD include biomarkers of brain amyloid-β (Aβ) protein deposition and of downstream neuronal degeneration or injury [4, 5]. However, these investigations require lumbar puncture (for cerebrospinal fluid analysis) or advanced neuroimaging techniques (positron emission tomography [PET] or structural magnetic resonance imaging [MRI]). Thus, the routine use of these biomarkers is limited because of the cost and invasive nature of the tests.

It is also becoming increasingly important to develop methods for accurate and early detection of AD. In 2022, 143 agents were in clinical trials for AD, with most (83.2%) being disease-modifying therapies, predominantly aimed at patients with preclinical AD, prodromal AD, or mild dementia due to AD [6]. As these medications enter clinical practice, there is a growing need for reliable and sensitive markers that would be accessible in community settings, making it possible to screen wider populations of patients that would benefit from further testing.

Olfactory dysfunction was reported in patients with AD nearly 50 years ago [7]. It has since been proven to be a common symptom present in 85–90% of the patients with AD [8, 9]. It is not only a common symptom, but also a very early sign of AD. Olfactory impairment is consistently found in patients with mild cognitive impairment (MCI) [10–12]. It is also present in patients with subjective cognitive decline (SCD) and is thought to precede cognitive symptoms for several years [13, 14]. However, studies involving patients with MCI and SCD often vary in terms of subtyping patients or do not provide information on the subtypes [11, 13]. Thus, it is difficult to apply these results in clinical practice, as it is likely that patients with disorders other than AD may have been included in the study samples [11].

Nevertheless, the value of olfactory testing is also supported by longitudinal studies, as olfactory dysfunction in healthy individuals was found to be associated with an increased risk of developing MCI on follow-up [15–18]. Furthermore, olfactory impairment is associated with an increased risk of conversion to dementia [19–22]. On the other hand, intact olfactory abilities are associated with a low likelihood of future dementia [23]. Although longitudinal studies have the same limitations pertaining to variable selection and lack of subtyping data for MCI, they confirm the value of further research on olfactory impairment as a marker of early AD.

Previous clinical findings are corroborated by pathological evidence. It has been reported that structures of the olfactory system (olfactory bulb, anterior olfactory nucleus, entorhinal, and transentorhinal areas) are affected by AD pathology in the early stages of the disease [24, 25]. Functional MRI and [^18^F]fluorodeoxyglucose PET studies have revealed structural and functional abnormalities in olfaction-related regions in the earliest stages of AD, and these changes were found to progress during the course of the disease [26–29].

Considering the evidence from previous studies, olfactory testing undoubtedly has the potential to be introduced into clinical practice and improve AD diagnostics if data are obtained using generally accepted AD diagnostic criteria and standardized assessment methods. In most studies, olfactory testing includes odor identification, and the odor discrimination ability of the patients has rarely been analyzed [13–23], even though both odor identification and odor discrimination tasks are considered to reflect higher processing of odors, that is impaired in the case of AD [30]. Moreover, performance on odor identification tests is heavily influenced by patients’ personal and cultural experiences and familiarity with different odors, making it impossible to use the same odor identification tests across different populations without adaptations [31]. Odor identification is also known to be influenced by a subject’s language abilities, making it difficult for researchers to interpret the results [32, 33]. Odor discrimination, although not completely independent of these factors, does not experience these limitations to the same extent as odor identification.

The aim of our study was to analyze odor discrimination in patients with early AD and explore its diagnostic qualities. We hypothesized that odor discrimination is impaired in the early stages of the disease and can reliably differentiate patients with AD, even those in prodromal stage, from subjects with CN.

## MATERIALS AND METHODS

### Participants

Thirty patients diagnosed with mild dementia due to Alzheimer’s disease (MD-AD), 30 with mild cognitive impairment due to Alzheimer’s disease (MCI-AD), and 30 elderly subjects with normal cognition (CN) were enrolled in the study.

The patients with MD-AD met the NIA/AA (National Institute on Aging-Alzheimer’s Association) criteria for probable Alzheimer’s disease [5] and had a Clinical Dementia Rating (CDR) of 1. All the patients were recruited from the memory clinic of Vilnius University hospital Santaros Klinikos. Probable AD was diagnosed by a specialist based on core clinical criteria with increased level of certainty, as all the patients had documented progressive cognitive decline [5]. Biomarker probability of AD etiology was intermediate, as all the patients had evidence of neuronal injury based on structural MRI that was performed as a standard clinical practice when diagnosing AD based on regulations by the Ministry of Health of The Republic of Lithuania [5]. Biomarkers of brain Aβ protein deposition were not available.

Patients with MCI-AD met the NIA/AA criteria for MCI due to AD [4] and had a CDR of 0.5. All the patients were recruited from the memory clinic of Vilnius University hospital Santaros Klinikos. MCI due to AD was diagnosed by a specialist when clinical and cognitive criteria were established and etiology of MCI was consistent with AD pathophysiological process based on exclusion of vascular, traumatic, medical causes of cognitive decline, and documented longitudinal decline in cognition [4]. Biomarker probability of AD etiology was intermediate, as all the patients had evidence of neuronal injury based on structural MRI that was performed as a standard clinical practice when diagnosing AD based on regulations by the Ministry of Health of The Republic of Lithuania [4]. Biomarkers of brain Aβ protein deposition were not available.

Elderly subjects with CN had no cognitive complaints, a CDR of 0, and no neurological disorders.

The exclusion criteria were as follows: central nervous system disorders other than MCI-AD and MD-AD, head trauma, cerebrovascular disorders (Hachinski Ischemic Score≥4), psychosis, depression (Geriatric Depression Scale score > 9), substance abuse, psychoactive medications, significant exposure to volatile substances affecting olfactory function, smoking, nasal surgery, and recent viral infections affecting olfactory function. The study was approved by the Vilnius Regional Bioethics Committee (Approval Number 2021/6-1355-830). Written informed consent was obtained from all the participants before participation in the study.

### Assessments of cognitive function

Global cognition was evaluated using the Mini-Mental State Examination (MMSE). The Alzheimer’s Disease Assessment Scale-Cognitive Subscale, version 13 (with additional delayed word recall and number cancellation tasks [ADAS-Cog 13], scores range: 0–85) was used for a detailed cognitive evaluation. Delayed recall was evaluated on a scale of 0 to 10 (number of words not recalled). The number cancellation task was evaluated using a scale from 0 to 5, with 0 indicating the best (≥30 correct responses) and 5 indicating the worst (0–5 correct responses) performance. Verbal fluency tests were also performed (PAS and animal naming), and the severity of cognitive impairment was evaluated using the Clinical Dementia Rating scale.

### Assessment of odor discrimination

Olfactory discrimination was evaluated by Sniffin’ Sticks tesst (Burghardt ®, Wedel, Germany); 16 triplets of odors were presented during the test. The participants were asked to identify which sample of the three had a different odor from the other two. The olfactory discrimination score was the number of correct responses out of 16.

The odors were presented in the order provided by the test instructions. Each odor was presented only once, for 3–4 s. The time interval between odors in the same triplet was 3 s. A time interval of 30 s was maintained between the sets of triplets. The subjects were instructed not to eat or drink anything for at least 15 min prior to testing. The examiner used odorless gloves, and the subjects wore a blindfold as per the test instructions.

### Data analysis

Statistical analysis was performed using IBM SPSS Statistics version 26.0. The Shapiro– Wilk test was used to check whether data distribution was normal. Differences between groups were tested using the Kruskal– Wallis test for numerical variables and two-tailed chi-square test for categorical variables. Spearman rank correlation coefficient was used to determine the correlation between the variables. Linear regression was used to analyze prediction of continuous dependent variables, and multinomial logistic regression was used to analyze prediction of categorical variables. The performance of the diagnostic tests was evaluated by receiver operating characteristic (ROC) curve analysis. A *p*-value of < 0.05 was considered significant.

## RESULTS

### Demographic and clinical characteristics

The demographic and clinical characteristics of the patients are presented in Table 1. The participants in the three groups showed no difference in sex distribution (chi-square test, *p* > 0.05). There were also no significant differences according to education, depressive symptoms (Geriatric Depression Scale results), and Hachinski ischemic score (for all, Kruskal– Wallis *p* > 0.05).

**Table 1:** Demographic and clinical characteristics of the participants

	CN	MCI-AD	MD-AD	Statistics (*χ*^2^(2)
	(*N* = 30)	(*N* = 30)	(*N* = 30)	/H(2), p)
Male (%) *	13 (43.33%)	13 (43.33%)	12 (40%)	0.09, 0.96
Years of education *	15 (3)	16 (2)	16 (3)	0.84, 0.66
Age **	74 (7)	72 (10)	78 (4)	16.8,<0.001
GDS *	5.5 (2)	5.5 (2)	5 (2)	0.08, 0.96
HIS *	1 (1)	1 (1)	1 (0)	2.09, 0.35

Data are presented as median and interquartile range unless specified otherwise. *The groups did not differ significantly. **MD-AD group differed significantly from the CN and MCI-AD groups. The CN and MCI-AD groups did not show any significant differences. MD-AD, mild dementia due to Alzheimer’s disease; MCI-AD, mild cognitive impairment due to Alzheimer’s disease; CN, normal cognition; GDS, Geriatric depression scale; HIS, Hachinski ischemic score.

Participants in the MD-AD group were older than those in the MCI-AD and CN groups (Kruskal– Wallis *p* < 0.05; post-hoc analysis revealed significant differences between the CN and MD-AD, MCI-AD, and MD-AD groups, and no significant difference between the CN and MCI-AD groups).

As expected, the performance on the cognitive tests was significantly different between all three groups (for all, Kruskal– Wallis *p* < 0.05, post-hoc analysis revealed significant differences among the three groups). The results of cognitive tests are presented in Table 2.

**Table 2:** Cognitive performance of the participants

	CN	MCI-AD	MD-AD	Statistics
	(*N* = 30)	(*N* = 30)	(*N* = 30)	(H(2), p)
MMSE *	29 (1)	26 (1)	22 (2)	79.46,<0.001
ADAS-Cog 13 *	10.83 (4.58)	20.84 (4.42)	29.34 (5.66)	69.5,<0.001
CDR Sum of Boxes *	0 (0)	2 (1)	5 (1)	82.67,<0.001
Fluency PAS *	37 (13)	28.5 (9)	21 (13)	29.79,<0.001
Fluency Animals *	20 (7)	13 (6)	10 (5)	42.22,<0.001

Data are presented as median and interquartile range. *All three groups differed significantly. MD-AD, mild dementia due to Alzheimer’s disease; MCI-AD, mild cognitive impairment due to Alzheimer’s disease; CN, normal cognition.

### Odor discrimination

**Fig. 1:**
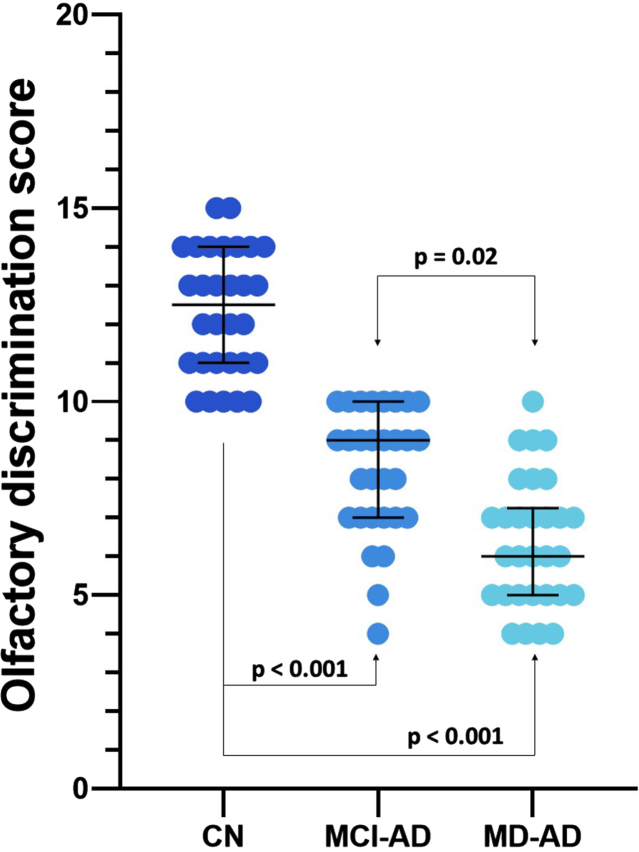
Olfactory discrimination scores of the three groups. Lines represent medians, error bars represent Interquartile ranges, and dots represent individual data points. MD-AD, mild dementia due to Alzheimer’s disease; MCI-AD, mild cognitive impairment due to Alzheimer’s disease; CN, normal cognition.

The olfactory discrimination scores differed significantly among the three groups (medians and interquartile ranges: CN 12.5 (3), MCI-AD 9 (3), MD-AD 6 (2); Kruskal– Wallis *p* < 0.05, post hoc analysis revealed significant differences between all three groups). The results of the odor discrimination test are presented in Fig. 1.

In the sample of all participants, the olfactory discrimination scores strongly correlated with the results of the MMSE and ADAS-Cog 13 (Spearman’s rho 0.78 and –0.77, respectively; *p* < 0.001). The olfactory discrimination scores also showed a strong correlation with the CDR sum of boxes (Spearman’s rho=–0.82; *p* < 0.001). The correlation between the olfactory discrimination scores and verbal fluency tests was also significant but at a moderate level (Spearman’s rho for PAS fluency = 0.64, for animal fluency = 0.64; *p* < 0.001 for both).

**Fig. 2:**
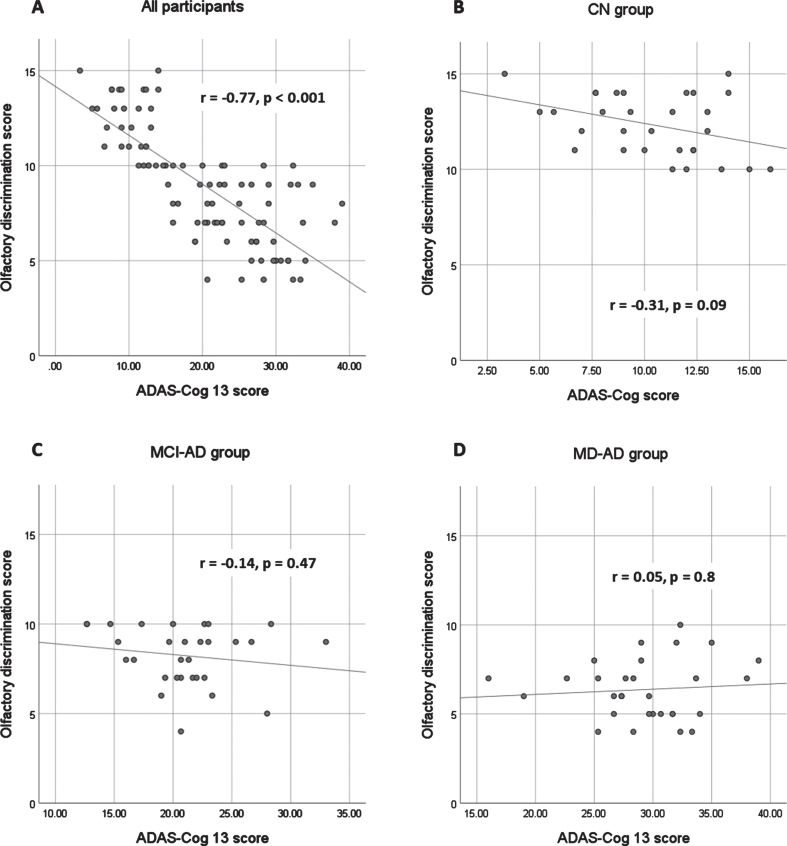
Relationship between olfactory discrimination scores and ADAS-Cog 13 results. ADAS-Cog 13, Alzheimer’s Disease Assessment Scale-Cognitive Subscale, version 13. MD-AD, mild dementia due to Alzheimer’s disease; MCI-AD, mild cognitive impairment due to Alzheimer’s disease; CN, normal cognition.

When analyzing the correlations in each group, no significant correlations were found between olfactory discrimination scores and cognitive test results in the MD-AD group. In the CN and MCI-AD groups, there were no significant correlations between odor discrimination and MMSE, ADAS-Cog 13, animal fluency, and CDR Sum of Boxes score. The correlation between olfactory discrimination scores and fluency PAS scores remained significant, although weak (Spearman’s Rho CN 0.45, MCI-AD 0.44; *p* < 0.05 in both cases). The relationship between the olfactory discrimination scores and ADAS-Cog 13 results is shown in Fig. 2.

The olfactory discrimination scores correlated significantly, although very weakly, with age when analyzing the entire sample (Spearman’s Rho –0.28; *p* = 0.008). There were no significant correlations between odor discrimination ability and age in the separate groups.

Multiple linear regression models, including age, sex, education, and cognitive test scores (MMSE, ADAS-Cog-13, CDR Sum of Boxes, and composite verbal fluency test score [VFT = PAS fluency+animal fluency]) as independent variables, were tested to determine whether they significantly predicted olfactory discrimination scores. The overall regression was statistically significant for all four models: model with MMSE R^2^ = 0.62, *F* = 35.05, *p* < 0.001; model with ADAS-Cog 13 R^2^ = 0.58, *F* = 29.43, *p* < 0.001; model with CDR Sum of Boxes R^2^ = 0.63, *F* = 36.25, *p* < 0.001; model with VFT R^2^ = 0.46, *F* = 17.72, *p* < 0.001. However, only cognitive test scores significantly predicted olfactory discrimination scores in each case (β for MMSE = 0.79, *p* < 0.001; for ADAS-Cog 13=–0.77, *p* < 0.001; for CDR Sum of Boxes=–0.8, *p* < 0.001; for VFT = 0.73; *p* < 0.001). None of the other predictors (age, sex, and education) significantly predicted the olfactory discrimination scores in any of the models (*p* > 0.05).

### Diagnostic characteristics of odor discrimination

**Fig. 3:**
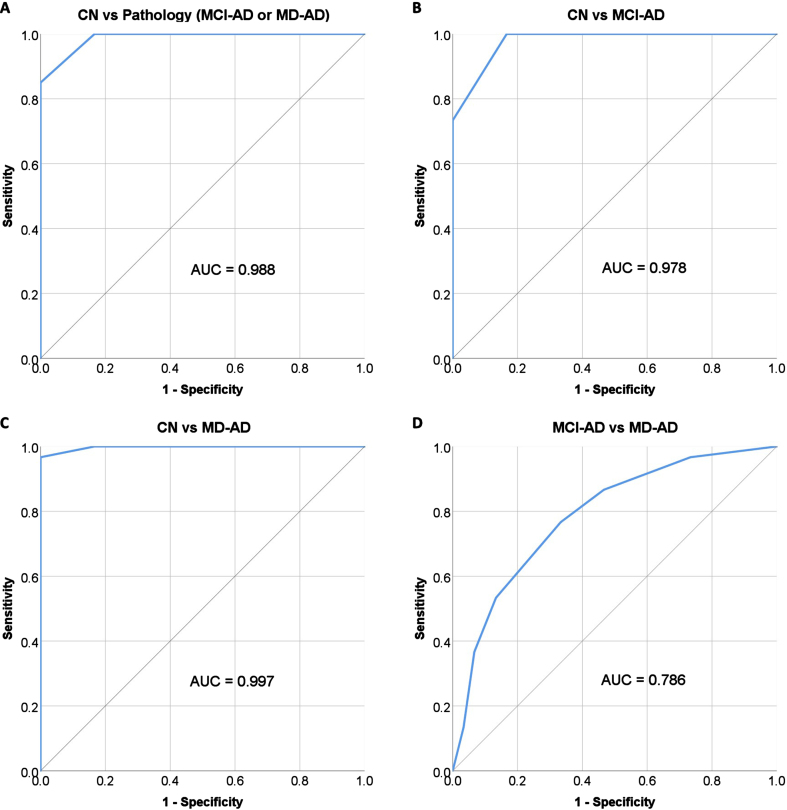
Performance of olfactory discrimination score in differentiating between participants with AD and CN. MD-AD, mild dementia due to Alzheimer’s disease; MCI-AD, mild cognitive impairment due to Alzheimer’s disease; CN, normal cognition.

ROC analysis was performed to evaluate the performance of the olfactory discrimination score in differentiating the CN group from AD patients (MCI-AD or MD-AD), the CN group from MCI-AD patients, the CN group from MD-AD patients, and MCI-AD patients from MD-AD patients. The ROC curves with areas under the curve (AUC) are shown in Fig. 3.

A cut-off score of≤10 correct responses was chosen for differentiating patients with AD (MCI-AD or MD-AD) from subjects with CN; the score had a sensitivity of 100% (95% confidence interval [CI], 94.04% to 100.00%) and specificity of 83.33% (95% CI, 65.28% to 94.36%). The negative predictive value was 100%, and the positive predictive value was 92.31% (95% CI, 84.35% –96.39%). The overall diagnostic accuracy was 94.44% (95% CI, 87.51% to 98.17%).

The diagnostic characteristics remained good when differentiating MCI-AD patients from subjects with CN. The same cut-off score of≤10 had a sensitivity of 100% (95% CI, 88.43% to 100.00%) and specificity of 83.33% (95% CI, 65.28% to 94.36%). The negative predictive value was 100% and the positive predictive value was 85.71% (95% CI, 72.94% to 93.03%). The overall diagnostic accuracy was 91.67% (95% CI, 81.61% to 97.24%).

Multinomial logistic regression was performed to analyze the relationship between the predictor variables and membership in the three groups (CN, MCI-AD, and MD-AD). First, a model with age, education, sex, and ADAS-Cog 13 scores as predictor variables was tested. The fit between the model containing only the intercept and the data improved with the addition of predictor variables (chi-square =139.66, *p* < 0.001; Nagelkerke R^2^ = 0.89). Pearson’s chi-square and deviance chi-square tests indicated that the model exhibited a good fit to the data (*p* > 0.05). The overall percentage of correctly classified cases using this model was 82.2% (CN, 93.3%; MCI-AD, 70%; MD-AD, 83.3%), with the ADAS-Cog 13 score as the strongest and most significant predictor (chi-square =122.65, *p* < 0.001).

The olfactory discrimination scores were included in the model. The model with age, education, sex, ADAS-Cog 13 scores, and olfactory discrimination scores also showed a significant improvement in fit over a null model (chi-square=158.11, *p* < 0.001; Nagelkerke R^2^ = 0.93). Pearson’s chi-square and Deviance chi-square tests indicated that the model exhibited a good fit to the data (*p* > 0.05). The overall percentage of correctly classified cases using this model was 92.2% (CN, 96.7%; MCI-AD, 86.7%; MD-AD, 93.3%), with the ADAS-Cog 13 and olfactory discrimination scores both being strong and significant predictors (chi-square=28.01 and 18.45, respectively, *p* < 0.001 for both).

## DISCUSSION

The levels of olfactory discrimination differed significantly among the three groups. We found that odor discrimination was impaired in the prodromal stage of AD (MCI-AD), and that the impairment became even more pronounced in patients with MD-AD. These findings are in accordance with the findings of previous studies, confirming the occurrence of olfactory impairment in the earliest stages of AD and the worsening of these changes as the disease progresses [10, 14].

The performance of the subjects in the current study was consistent with that reported in previous studies. According to normative data, the scores of healthy elderly subjects on odor discrimination tests range from 10.66±2.5 to 13.80±0.77 [34, 35]. The results of the CN group in the current study were within this range. The performance of patients with early AD was also similar to that reported in previous studies, where odor discrimination scores of the patients with MCI ranged from 7.9±3.2 to 10.3±2.6 and scores of the patients with AD ranged from 5.6±3.8 to 9.6±2.3 [12, 36, 37].

Of note, the study reporting the best odor discrimination results in the MCI group (10.3±2.6) did not evaluate the subtype of MCI [37]. In order to apply the results in clinical practice, researchers must ensure that the study subjects meet the criteria for MCI due to AD in order to avoid including patients with disorders other than AD.

Olfactory discrimination scores showed a strong and significant correlation with the results of the cognitive tests in our study. Linear regression analysis demonstrated a significant relationship between these elements, with age, sex, and education having no significant influence on the odor discrimination score. These findings confirm that olfactory deficits in patients with AD are associated with the processes of the disease itself and cannot be explained by other factors known to affect olfaction in the general population, such as age and sex [34, 35]. These results are not unexpected, since structural and functional abnormalities have been detected in olfaction-related cortical regions in patients with AD [26–29].

The diagnostic qualities of olfactory tests have been examined in previous studies; however, information on the diagnostic qualities of olfactory discrimination tests specifically is still lacking as olfactory identification is usually the test of choice [12–23, 36, 37]. Our study yielded promising results, especially for improving the diagnosis of prodromal AD (MCI-AD).

In the current study, odor discrimination demonstrated excellent capabilities in differentiating patients with early AD (MCI-AD or MD-AD) from healthy controls (AUC = 0.988) and in differentiating patients with prodromal AD (MCI-AD) from healthy controls (AUC = 0.978). This is in accordance to findings from previous studies, where odor discrimination was found to be a significant and more reliable predictor of future cognitive decline, than odor identification [38]. However, other authors did not find odor discrimination to be superior to odor identification, even though both of them performed better then odor threshold in differentiating patients with AD and patients with MCI from cognitively normal participants [39].

Furthermore, the inclusion of olfactory discrimination scores into the multinomial logistic regression models improved the overall classification accuracy by 10% (the accuracy of classification into three groups [CN, MCI-AD, and MD-AD] improved from 82.2% to 92.2%). The correct classification of MCI-AD cases improved the most, by 16.7% (from 70% to 86.7%). This finding is important since prodromal AD (MCI-AD) might be difficult to diagnose, especially in primary care settings. According to a survey by the Alzheimer’s Association, nearly two-thirds of primary care physicians (65%) said they were comfortable diagnosing MCI, while less than half (49%) reported being comfortable diagnosing MCI-AD [38]. Objective olfactory testing may be very useful for improving diagnostic certainty. Especially the certainty in differentiation of MCI from normal aging, which was the most frequently cited challenge when making the diagnosis (72%) [40].

Although odor discrimination had excellent diagnostic qualities in differentiating patients with early AD from healthy controls, its performance in differentiating between prodromal AD (MCI-AD) and patients with mild dementia (MD-AD) was not as good (AUC = 0.786) as expected in the current study. Similar results were found in recent studies on odor identification (SST12 test had an AUC of 0.741 for differentiation of MCI-AD from MD-AD) [41]. This is most likely because olfactory impairment occurs early in the course of the disease and is pronounced even in the prodromal stage of AD, making it not suitable for monitoring disease progression.

Early involvement of the olfactory system in the course of AD, which was once again confirmed by the findings of the current study, has recently led to research on olfactory training as a treatment method. Olfactory training was found to improve cognitive functioning in patients with dementia [42]. Moreover, olfactory training had a positive effect on frontal lobe activation in response to odors and increased the cortical thickness of the hippocampus [43, 44]. Even though the data are still limited at this time, these findings warrant further research on olfaction in AD.

The present study has several limitations. First, the cross-sectional design limits the accuracy of the conclusions regarding the progression of changes during the course of AD. Also, although the results regarding diagnostic properties of the odor discrimination test are encouraging, they need to be confirmed in further studies, especially estimations of negative and positive predictive values of the test. Second, CSF analysis and PET were not used, and including these modalities would have helped us analyze the relationship between olfactory changes and brain Aβ deposition and neuronal degeneration. Moreover, CSF and PET biomarkers would help in confirming the diagnosis. In the current study, all the participants met criteria for probable AD or MCI consistent with AD pathophysiological process [4, 5]. However, CSF and PET biomarkers would be helpful in increasing the level of certainty. Third, although the differences were significant in our small sample, studies involving larger sample sizes would help confirm these findings.

In conclusion, the current study showed that odor discrimination is already impaired in the prodromal stage of AD and that these changes progress during the course of the disease. Olfactory discrimination testing has good diagnostic qualities and can help clinicians accurately diagnose early AD, serving as a simple, noninvasive, affordable, and reliable marker.

## Data Availability

Data supporting the findings of this study are available upon request from the corresponding author.
